# Human Chorionic Gonadotropin as a Pivotal Endocrine Immune Regulator Initiating and Preserving Fetal Tolerance

**DOI:** 10.3390/ijms18102166

**Published:** 2017-10-17

**Authors:** Anne Schumacher

**Affiliations:** Experimental Obstetrics and Gynecology, Medical Faculty, GC-I^3^, Otto-von-Guericke University Magdeburg, 39108 Magdeburg, Germany; anne.schumacher@med.ovgu.de; Tel.: +49-391-6717510

**Keywords:** human chorionic gonadotropin, dendritic cells, regulatory T cells, B cells, fetal tolerance

## Abstract

The pregnancy hormone, human chorionic gonadotropin (hCG), is crucially involved in processes such as implantation and placentation, two milestones of pregnancy whose successful progress is a prerequisite for adequate fetal growth. Moreover, hCG determines fetal fate by regulating maternal innate and adaptive immune responses allowing the acceptance of the foreign fetal antigens. As one of the first signals provided by the embryo to its mother, hCG has the potential to regulate very early pregnancy-driven immune responses, allowing the establishment and preservation of fetal tolerance. This mini review focuses on how hCG modulates the adaptive arm of the immune system including dendritic cells as key regulators of adaptive immune responses.

## 1. Introduction

In 2012, Laurence A. Cole published a comprehensive review in which he designated the pregnancy hormone human chorionic gonadotropin (hCG) as a wonder of today’s science [1]. Indeed, hCG not only exhibits unique biochemical peculiarities but also possesses a multitude of biological functions including more activities than just maintaining luteal steroidogenesis. Besides supporting the implantation and placentation process, hCG is best known for its immunological properties. Being the first embryo-derived signal, hCG is suggested to profoundly influence early pregnancy-driven maternal immune responses, thereby ensuring fetal tolerance induction. By increasing the number of uterine natural killer cells, hCG contributes to a proper remodeling of the maternal spiral arteries which guarantees a sufficient nourishment of the fetus [2]. Moreover, hCG acts on the complement system, regulates apoptosis through the Fas/Fas-ligand system and modulates the balance between inflammatory type 1 T helper (TH) cells and anti-inflammatory type 2 TH cells [3,4], all of which are mechanisms that are critical for embryo survival. Furthermore, hCG affects fetal well-being by regulating the phenotype and functionality of dendritic cells (DCs), regulatory T (Treg) cells and B cells.

## 2. Human Chorionic Gonadotropin—Inducer of Tolerogenic Dendritic Cells?

DCs are key regulators of immune responses due to their prominent function as intermediaries between the innate and adaptive arm of the immune system. Depending on their maturation state and the type of cytokines they produce, DCs are capable of either driving immunity or inducing tolerance. In the prevention of autoimmunity or allograft rejection, factors promoting a tolerogenic DC phenotype and thereby dampening undesired immune responses are highly appreciated, whereas for inducing anti-cancer immunity they are counterproductive. hCG-mediated DC regulation has been suggested to play a role in all three immunological situations. Interestingly, hCG, in addition to its secretion by the placenta, is ectopically expressed by a variety of tumors and its production is associated with poor prognosis. Similar to its function during pregnancy, hCG supports tumorigenesis by promoting angiogenesis and by generating tolerogenic DCs through activation of indoleamine 2,3-dioxygenase (IDO) expression [5]. IDO is a rate-limiting enzyme for tryptophan degradation. As tryptophan is an essential amino acid for T cells, the depletion of local tryptophan by IDO-expressing DCs forces proliferation arrest and anergy in T cells [6] and impairs anti-tumor immunity. On the other hand, DCs can be used as targets to develop anti-cancer vaccines towards hCG-sensitive tumors by exposing DCs to hCG and subsequently inducing hCG-specific proliferative and cytotoxic T-cell responses [7]. In autoimmunity, hCG has been identified as a beneficial factor for disease prevention. After repeated hCG injections in non-obese diabetic (NOD) mice, an induction of IDO in DCs could be observed that resulted in an inhibition of autoreactive T cells and the prevention of disease onset [8]. Before and during pregnancy, hCG seems to affect different aspects of DC biology. For instance, it has been suggested that hCG may attract DCs from the circulation into the ovary, where these ovarian DCs are supposed to contribute to the ovulation process [9,10]. Additionally, hCG was shown to decrease the proportion of mature ovarian DCs, proposing that hCG particularly increases the abundance of immature DCs in the ovary [11]. After pregnancy is established, hCG may differentially influence the local and peripheral DC pool. Several studies reported various outcomes after hCG treatment of DCs obtained from distinct tissue sites. Segerer and colleagues generated immature human DCs from blood-derived monocytes and induced differentiation in the presence of hCG. The hormone inhibited the up-regulation of maturation markers as well as the T cell stimulatory capacity of the DCs retaining a tolerogenic phenotype in these cells [12]. In sharp contrast, Yoshimura and colleagues found that hCG up-regulated maturation markers on peripheral blood DCs, stimulated the secretion of inflammatory cytokines and enhanced their ability to activate T cells [13]. In the murine system, we and others demonstrated an inhibitory effect of hCG on bone marrow-derived DCs as well as on peripheral and local (decidual) DCs supporting the idea that hCG supports a tolerogenic rather than an immunogenic DC phenotype [14,15,16]. However, it can be speculated that peripheral and local DCs need to be differentially regulated during pregnancy to allow immune tolerance towards fetal alloantigens on one hand and immune competence towards pathogens on the other. Based on the above-mentioned findings, it can be assumed that hCG is involved in this regulation process.

## 3. Human Chorionic Gonadotropin—Supporter of Baby’s Best Friends

Recently, we introduced Treg cells as baby’s best friends due to their great potential to protect the fetal tissue from maternal immune attacks [17]. Their indispensable role for fetal tolerance induction has been proven by a multitude of human and murine studies. Particularly, a sufficient number and adequate functionality of this unique T cell population at early pregnancy stages are a prerequisite for proper embryo implantation [18,19,20] and the prevention of fetal rejection [19,21,22,23,24]. By contrast, depletion of Treg cells at later pregnancy stages could not be associated with pregnancy disturbances, suggesting that their participation in pregnancy maintenance is less important [19]. However, these previous findings need to be confirmed in future studies. Human and murine Treg cells are highly susceptible to a regulation by pregnancy hormones and there are several studies showing an influence of progesterone and estrogen on the number and activity of Treg cells [25,26,27,28]. First evidence for an hCG-mediated modulation of Treg cells was already introduced in the 1980s when Treg cells were still named “T suppressor cells”. At this particular time, Fuchs and colleagues proved that hCG is able to induce human and murine T suppressor cells capable of depressing a polyclonal antibody response induced by different B cell mitogens [29,30]. Notably, the hCG-driven effect on T lymphocytes seems to be sex-dependent as hCG only induced T suppressor cells in lymphocytes from females but not from males. Fuchs and colleagues concluded that a gene(s) located on the Y-chromosome may exert a regulatory function and thereby prevent the hormone from inducing T suppressor cells [31]. Some years later, hCG has been proposed to possess a stimulating effect on precursors of T suppressor cells, without affecting mature specific T suppressor cells [32]. In 2009, we performed a human study where we compared patients suffering from spontaneous abortions or extra uterine pregnancies with normal pregnant women. Our analysis revealed that patients with pregnancy complications not only had significantly reduced hCG levels but also had significant lower Treg cell levels at the fetal–maternal interface. This finding led us to assume that hCG may function as an attractor for Treg cells into the fetal–maternal interface and indeed we confirmed that hCG-producing trophoblast cells efficiently attract Treg cells and interact with each other [33]. In a follow-up study, we showed that in addition to its function as a Treg cell attractor, hCG has the potential to provoke the conversion of non-Treg cells into Treg cells [34]. In line with this, a recent human study reported that the percentages of CCR4^+^Foxp3^+^ Treg cells and TGF-β-expressing Foxp3^+^ Treg cells increased after stimulating naïve T cells with anti-CD3/CD28 and hCG [20]. The authors proposed that hCG inhibits the expression of phosphorylated AKT and phosphorylated ERK (extracellular signal-regulated kinases) that are required to induce Treg cell differentiation [35]. Two other studies showed that hCG is able to increase the number of Treg cells within human mononuclear cells [36,37]. Studies performed in the murine system suggested that hCG may protect fetal rejection by increasing the number and function of Treg cells. Repeated hCG injections during the pre- and peri-implantation phase into abortion-prone females significantly increased the peripheral and local Treg cell numbers and augmented the Treg cell-suppressive capacity towards T effector cells. The hCG-induced changes resulted in a significant diminution of the fetal rejection rate [15]. However, hCG injections during late gestation have to be taken with caution. Although it is suggested that hCG applications in late pregnancy stages increase Treg cell number and prevent endotoxin-induced preterm birth, it has also been shown to result in dystocia and fetal compromise and may therefore not be suitable for preterm birth prevention in humans [38]. On the other hand, hCG might be useful in the treatment of unexplained recurrent spontaneous abortion (URSA). Here, a combination therapy of hCG and immunoglobulin shifted the TH17/Treg ratio in favor of the Treg cells and thereby restored the disturbed TH17/Treg balance which had been detected in URSA patients and patients suffering from other pregnancy complications [39,40]. Interestingly, hCG and immunoglobulin treatment not only affected the TH17/Treg ratio but also decreased TH17-related cytokines and elevated Treg-related cytokines proposing an effect of the treatment on both number and function of TH17 and Treg cells [39]. In a more detailed study, the role of CG and synthetic CG β-subunit oligopeptides on TH17 and Treg cell differentiation was investigated. Both CG and synthetic oligopeptides elevated the level of Treg cells and their functional activity. Moreover, they prevented the differentiation of TH0 cells into TH17 cells and significantly suppressed the activity of this inflammatory TH subset [41]. Hence, synthetic CG β-subunit oligopeptides in addition to the whole molecule may be suitable for the treatment of spontaneous abortion as they maintain an adequate Treg/TH17 balance shown to be pivotal for successful pregnancy outcomes.

Based on these findings, hCG is suggested to promote Treg cells either by direct binding to its receptor on T cells and/or by indirect pathways. These indirect pathways may include hCG-driven changes in the phenotype and function of other immune cell populations such as DCs as well as changes in the levels of the steroid hormones progesterone and estrogen. By augmenting the levels of both steroid hormones, hCG is able to further enhance Treg cells.

## 4. Human Chorionic Gonadotropin—Friend or Foe for B Cell-Mediated Fetal Tolerance

While much attention has been paid in the past to the different T cell subsets during pregnancy, the tremendous contribution of B cells to fetal tolerance has been overlooked for a long time. Now it has become more and more evident that different B cell subpopulations undergo dramatic changes during pregnancy progression. For instance, it has been demonstrated that the frequencies of B1 B cells decrease whereas the number of conventional B2 B cells remains relatively constant [42]. Moreover, the levels of B cells specific for paternal antigens are partially deleted during pregnancy, which contributes to the success of the fetal allograft [43]. As a major function, B cells can produce either pregnancy-protective or pregnancy-destructive antibodies and by doing so they have the potential to determine fetal fate [44,45]. With respect to a regulation by hCG, the majority of studies focused on the potential of B cells to produce anti-hCG antibodies that can be used in anti-cancer treatments [46] and fertility preservation [47]. However, very little information is provided about if and how hCG modulates B cell number and activity and thereby affects fetal tolerance, induction and maintenance. When studying the participation of B1a B cells in pregnancy disturbances, we found a significant elevated number of this B cell subpopulation in pre-eclamptic patients in the third trimester when compared to normal pregnant women. Interestingly, the B1a B cell increase was associated with pathologically elevated hCG levels in those patients which led us to assume that hCG may drive B1a B cell augmentation. Indeed, we could confirm that not only ~95% of the B1a B cells expressed the LH/CG (luteinizing hormone/CG) receptor but these cells also expand on hCG stimulation in a lymphocyte culture. Furthermore, we proved that isolated B1a B cells are able to produce autoantibodies that may provoke pre-eclampsia-associated symptoms [48]. In agreement, Kalkunte and colleagues reported higher serum hCG levels in pre-eclamptic patients and suggested that an altered glycosylation pattern and/or the presence of sialyl Lewis antigens on hCG may influence the recruitment and/or expansion of tolerance-imparting immune cells [49]. Hence, B1a B cells may represent a target of hCG in this specific pregnancy disorder and hCG plays a rather detrimental role in this context. On the other hand, an adequate hCG rise in the first trimester may induce fetal tolerance by fostering the generation and functionality of regulatory B (Breg) cells. Similar to Treg cells, Breg cells can efficiently suppress other immune cell populations and thereby dampen undesired immune responses towards organ and fetal allografts as well as in autoimmunity [50]. Our previous studies revealed a significantly diminished number of Breg cells in patients suffering from spontaneous abortion as compared to normal pregnant women, highlighting the importance of Breg cells for pregnancy success [51]. Moreover, our results indicated an effect of hCG on Breg cell number and activity. hCG not only increased the number of Breg cells but also boosted the production of interleukin-10 (IL-10), the hallmark of Breg cells [51,52]. Furthermore, hCG seems to influence the generation of plasma cells as well as their ability to produce a specific type of pregnancy-protective antibody. Hammarström and colleagues showed that hCG depressed the formation of plasma cells [53] and Cocchiarra and colleagues found an inhibited IgG production with high hCG concentrations in vitro, and with low hCG concentrations when cells were obtained from patients that had been treated with hCG in vivo [54]. Notably, we observed that hCG did not augment galactosylation, sialylation or fucosylation of IgG subclasses in their Fc region. However, hCG induced the synthesis of asymmetrically glycosylated antibodies in their Fab region [52]. As asymmetrical antibodies were strongly associated with pregnancy success [55], we suggest this process to be one important mechanism through which hCG positively influences B-cell mediated fetal tolerance. Additionally, the capability of hCG to promote Breg cells further contributes to the establishment of fetal tolerance during early pregnancy.

## 5. Human Chorionic Gonadotropin—Does the Source and Concentration Matter for Its Immune Regulatory Properties?

hCG comes in five different flavors, namely regular hCG, sulfated hCG, hyperglycosylated hCG, free hCG β-subunit and hyperglycosylated free hCG β-subunit, with each variant possessing unique biological functions [1]. All variants are produced during pregnancy and can be detected and purified from the urine of pregnant women. Urine-hCG (uhCG) preparations (e.g., Pregnyl, Profasi and Novarel) together with recombinant hCG (rhCG) preparations (e.g., Ovitrelle) are routinely used in the clinic for final oocyte maturation in patients undergoing artificial reproductive techniques (ART) [56]. However, both preparations differ significantly in various aspects. While uhCG preparations contain a heterogenous mixture of intact, nicked and cleaved hCG molecules, rhCG preparations are composed of only intact hCG molecules. Furthermore, the carbohydrate structure of uhCG slightly differs from the one of rhCG and uhCG preparations often contain a significant amount of non-hCG proteins and show high batch-to-batch variations [57]. These differences may partly account for the disparities in the efficacy of both hCG preparations that have been indicated in several studies [56], and may provide explanations for potential distinct immunological properties. To study the ability to regulate immune responses, both preparations were employed in in vivo and in vitro studies in the past. Unfortunately, some authors did not clearly indicate whether they used uhCG or rhCG, impeding the comparison between both preparations. Moreover, the hCG concentrations applied as well as the application frequency strongly differ between the various studies. Nevertheless, there are indications for concentration-dependent effects of hCG on DCs, Treg cells and B cells. Ueno and colleagues isolated splenic CD11c^+^ DCs from NOD mice and treated the cells with 200 or 400 IU of rhCG. Both hCG concentrations up-regulated IDO expression in DCs in vitro. Furthermore, DCs were isolated from the spleen of NOD mice that had received one single hCG injection at different concentrations (100, 200, 400 and 1000 IU). Interestingly, IDO expression in vivo was detectable in DCs 24 h after injection with 100 and 200 IU rhCG, and almost not detectable when higher concentrations were used [8]. Another study investigated the influence of 10 and 50 IU of hCG on murine T and B cells. While 10 IU of hCG selectively activated the B cells and failed to affect T cell functionality in vivo, 50 IU of hCG did not alter B cells but significantly suppressed T cell activity. This suggests a differential effect of high and low hCG concentrations on different immune cell populations [58]. Our own analysis revealed that both uhCG and rhCG had the potential to induce human Treg cells and to hamper maturation of murine DCs [16,34]. Although no concentration-dependency could be demonstrated for the effect of hCG on T cells, there was a tendency for a stronger impact on DCs with increasing concentrations. Moreover, in our hands, it seemed that rhCG has a stronger effect on both immune cell types. By contrast, Shirshev and colleagues observed an inhibitory effect of 10 IU of uhCG on the generation of natural Treg cells in human thymocyte cultures. This effect was abolished in the presence of 100 IU of uhCG [59]. Based on these findings, it is suggested that both the hCG preparation and the concentration used in ART may influence fetal tolerance induction and thereby determine pregnancy outcome. However, it cannot be assumed that much helps a lot, which may question the applied hCG doses. Finally, the immune regulatory potential of each hCG variant has to be clarified in the future.

## 6. Conclusions

**Figure 1:**
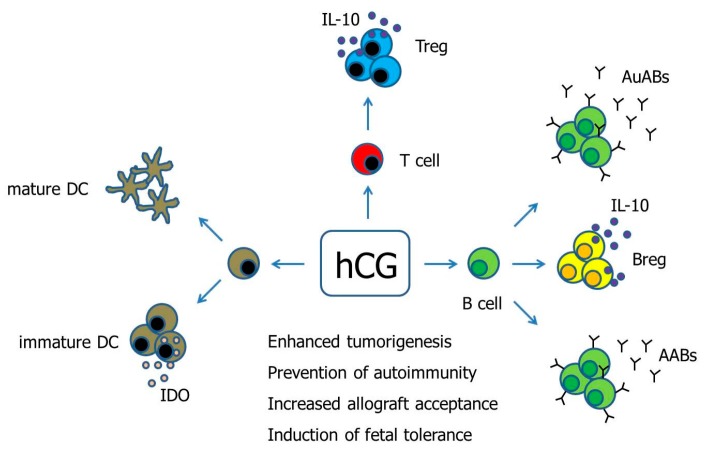
Hypothetical scenario proposing pathways of hCG-mediated immune modulation. Human Chorionic gonadotropin (hCG) has been suggested to differentially regulate DCs depending on the DC tissue-type. Moreover, hCG drives the conversion of conventional T cells into fully functional Treg cells. The effect of hCG on B cells seems to be manifold as the hormone can induce both the production of immunogenic autoantibodies as well as tolerogenic asymmetric antibodies, and can additionally provoke the generation of suppressive Breg cells. AABs—Asymmetric antibodies, AuABs—Autoreactive antibodies, Breg—regulatory B cell, DC—Dendritic cell, IDO—Indoleamine 2,3-dioxygenase, IL-10—Interleukin-10, Treg—regulatory T cells.

This mini review discussed the potential of hCG to modulate DCs, Treg cells and B cells, and consequently the effects on tumorigenesis, autoimmunity and fetal tolerance. Whereas the influence of hCG on DCs and B cells is still a matter of debate and seems to highly depend on the tissue origin and subpopulation, it is generally accepted that hCG promotes the generation and function of Treg cells (see Figure 1). Finally, to improve the clinical application of hCG, further research is needed to clarify whether the used doses are appropriate and whether one hCG preparation should be preferred over the other.
